# Supplementary material for: Comparative Efficacy of Subcutaneous Versus Intravenous Interleukin 12/23 Inhibitors for the Remission of Moderate to Severe Crohn’s Disease: A Systematic Review and Meta-Analysis
Source: Biomedicines. 2025 Mar 12;13(3):702. doi: 10.3390/biomedicines13030702 (PMC11940749; doi:10.3390/biomedicines13030702)
Supplement: Supplementary file 1 [file biomedicines-13-00702-s001.zip › Search strategy.pdf]

## Search Strategy

### PubMed search

**Search #1 MeSH terms for Interleukin 12/23 inhibitors (n= 83)**

"Interleukin Inhibitor"

**Search #2 Keywords for Interleukin 12/23 inhibitors (n= 369,064)**

"Interleukin Inhibitor" OR "Monoclonal antibody" OR "Monoclonal antibodies" OR "Biologics" OR "Ustekinumab" OR "Stelara" OR "Wezlana" OR "CNTO-1275" OR "CNTO1275" OR "anti-IL-12" OR "anti-IL-23" OR "Anti-IL-12/23 Therapy" OR "IL-12/23 Blocker" OR "IL-12/23 Inhibitor" OR "Guselkumab" OR "Tremfya" OR "Risankizumab" OR "Skyrizi" OR "Brazikumab" OR "Mirikizumab" OR "Omvoh"

**Search #3 combined search for Interleukin 12/23 inhibitors (n=51)**

**Search #4 MeSH terms for Crohn's Disease (n=46,344)**

"Crohn's Disease"

**Search #5 keywords for Crohn's Disease (n=74,052)**

"Crohn's Disease" OR "Crohns Disease" OR "Crohn Disease" OR "Crohn's Enteritis" OR "Regional Enteritis" OR "Ileocolitis" OR "Ileitis, Terminal" OR "Terminal Ileitis" OR "Ileitis, Regional" OR "Regional Ileitides" OR "Regional Ileitis" OR "Enteritis, Granulomatous" OR "Granulomatous Enteritis" OR "Refractory Crohn's Disease" OR "Severe Crohn's Disease" OR "Mild-to-Moderate Crohn's Disease"

**Search #6 combined search for Crohn's Disease (n= 46,344)**

**Search #7 MeSH terms for route of administration (n= 115,938)**

"Intravenous Infusions" OR "Subcutaneous Injection"

**Search #8 Keywords for route of administration (n= 3,138,188 )**

"Subcutaneous" OR "SC" OR "SQ" OR "SubQ" OR "Sub-Q" OR "Subcut" OR "SubC" OR "Subcu" OR "Hypodermic" OR "Hypodermal" OR "Intracutaneous" OR "Intravenous" OR "Drip" OR "Endovenous" OR "IV" OR "Venous" OR "Infusion"

**Search #9 – combined search for route of administration (n= 109,596)**

**Search #10- combined search (n= 1,140)**

## Cochrane Central Register of Controlled Trials (CENTRAL) search

### Search #1 Search terms for monoclonal antibody therapies (n=68695)

"Monoclonal antibody" OR "Monoclonal antibodies" OR "Biologics" OR "Ustekinumab" OR "Stelara" OR "Wezlana" OR "CNTO-1275" OR "CNTO1275" OR "anti-IL-12" OR "anti-IL-23" OR "Anti-IL-12/23 Therapy" OR "IL-12/23 Blocker" OR "IL-12/23 Inhibitor" OR "Guselkumab" OR "Tremfya" OR "Risankizumab" OR "Skyrizi" OR "Brazikumab" OR "Mirikizumab" OR "Omvoh"

### Search #2 Search terms for Crohn's Disease (n=32011)

"Crohn's Disease" OR "Crohns Disease" OR "Crohn Disease" OR "Crohn's Enteritis" OR "Regional Enteritis" OR "Ileocolitis" OR "Ileitis, Terminal" OR "Terminal Ileitis" OR "Ileitis, Regional" OR "Regional Ileitides" OR "Regional Ileitis" OR "Enteritis, Granulomatous" OR "Granulomatous Enteritis" OR "Refractory Crohn's Disease" OR "Severe Crohn's Disease" OR "Mild-to-Moderate Crohn's Disease"

### Search #3 Search terms for route of administration (n=286641)

"Subcutaneous" OR "SC" OR "SQ" OR "SubQ" OR "Sub-Q" OR "Subcut" OR "SubC" OR "Subcu" OR "Hypodermic" OR "Hypodermal" OR "Intracutaneous" OR "Intravenous" OR "Drip" OR "Endovenous" OR "IV" OR "Venous" OR "Infusion"

### Search #4 - #1 AND #2 AND #3 (n= 1322)

**Cumulative Index to Nursing and Allied Health Literature (CINAHL)**

**Search #1 Search terms for monoclonal antibody therapies (n=36,229)**
"Monoclonal antibody" OR "Monoclonal antibodies" OR "Biologics" OR "Ustekinumab" OR "Stelara" OR "Wezlana" OR "CNTO-1275" OR "CNTO1275" OR "anti-IL-12" OR "anti-IL-23" OR "Anti-IL-12/23 Therapy" OR "IL-12/23 Blocker" OR "IL-12/23 Inhibitor" OR "Guselkumab" OR "Tremfya" OR "Risankizumab" OR "Skyrizi" OR "Brazikumab" OR "Mirikizumab" OR "Omvoh"

**Search #2 Search terms for Crohn's Disease (n=11,778)**

"Crohn's Disease" OR "Crohns Disease" OR "Crohn Disease" OR "Crohn's Enteritis" OR "Regional Enteritis" OR "Ileocolitis" OR "Ileitis, Terminal" OR "Terminal Ileitis" OR "Ileitis, Regional" OR "Regional Ileitides" OR "Regional Ileitis" OR "Enteritis, Granulomatous" OR "Granulomatous Enteritis" OR "Refractory Crohn's Disease" OR "Severe Crohn's Disease" OR "Mild-to-Moderate Crohn's Disease"

**Search #3 Search terms for route of administration (n=262,062))**

"Subcutaneous" OR "SC" OR "SQ" OR "SubQ" OR "Sub-Q" OR "Subcut" OR "SubC" OR "Subcu" OR "Hypodermic" OR "Hypodermal" OR "Intracutaneous" OR "Intravenous" OR "Drip" OR "Endovenous" OR "IV" OR "Venous" OR "Infusion"

**Search #4 - #1 AND #2 AND #3 (n= 166)**

## SCOPUS search

### Search #1 Search terms for monoclonal antibody therapies (n=482,459)

“Monoclonal antibody” OR “Monoclonal antibodies” OR “Biologics” OR “Ustekinumab” OR “Stelara” OR “Wezlana” OR “CNTO-1275” OR “CNTO1275” OR “anti-IL-12" OR “anti-IL-23” OR “Anti-IL-12/23 Therapy” OR “IL-12/23 Blocker” OR “IL-12/23 Inhibitor” OR “Guselkumab” OR “Tremfya” OR “Risankizumab” OR “Skyrizi” OR “Brazikumab” OR “Mirikizumab” OR “Omvoh”

### Search #2 Search terms for Crohn’s Disease (n=101,077)

“Crohn's Disease” OR “Crohns Disease” OR “Crohn Disease” OR “Crohn’s Enteritis” OR “Regional Enteritis” OR “Ileocolitis” OR “Ileitis, Terminal” OR “Terminal Ileitis” OR “Ileitis, Regional” OR “Regional Ileitides” OR “Regional Ileitis” OR “Enteritis, Granulomatous” OR “Granulomatous Enteritis” OR “Refractory Crohn’s Disease” OR “Severe Crohn’s Disease” OR “Mild-to-Moderate Crohn’s Disease”

### Search #3 Search terms for route of administration (n=2,611,484)

“Subcutaneous” OR “SC” OR “SQ” OR “SubQ” OR “Sub-Q” OR “Subcut” OR “SubC” OR “Subcu” OR “Hypodermic” OR “Hypodermal” OR “Intracutaneous” OR “Intravenous” OR “Drip” OR “Endovenous” OR “IV” OR “Venous” OR “Infusion”

### Search #4 - #1 AND #2 AND #3 (n= 1,483)

## bioRxiv Search

**Search #1 Search terms for monoclonal antibody therapies (n=109)**
"Ustekinumab" OR "Stelara" OR "Wezlana" OR "Guselkumab" OR "Tremfya" OR "Risankizumab" OR "Skyrizi" OR "Brazikumab" OR "Mirikizumab" OR "Omvoh"

**Search #2 Search terms for Crohn's Disease (n=150,644)**

"Crohn's Disease" OR "Crohns Disease" OR "Crohn Disease"

**Search #3 Search terms for route of administration (n=134,176)**

"Subcutaneous" OR "SC" OR "Intravenous" OR "Drip" OR "IV" OR "Venous" OR "Infusion"

**Search #4 - #1 AND #2 AND #3 (n= 9)**

**MedRxiv- the preprint server for Health Sciences**

**Search #1 Search terms for monoclonal antibody therapies (n=106)**
“Ustekinumab” OR “Stelara” OR “Wezlana” OR “Guselkumab” OR “Tremfya” OR “Risankizumab” OR “Skyrizi” OR “Brazikumab” OR “Mirikizumab” OR “Omvoh”

**Search #2 Search terms for Crohn’s Disease (n=53,011**)

“Crohn's Disease” OR “Crohns Disease” OR “Crohn Disease”

**Search #3 Search terms for route of administration (n=26,835)**

“Subcutaneous” OR “SC” OR “Intravenous” OR “Drip” OR “Endovenous” OR “IV” OR “Venous” OR “Infusion”

**Search #4 - #1 AND #2 AND #3 (n= 9)**
