# Supplementary material for: Comparative Efficacy of Subcutaneous Versus Intravenous Interleukin 12/23 Inhibitors for the Remission of Moderate to Severe Crohn’s Disease: A Systematic Review and Meta-Analysis
Source: Biomedicines. 2025 Mar 12;13(3):702. doi: 10.3390/biomedicines13030702 (PMC11940749; doi:10.3390/biomedicines13030702)
Supplement: Supplementary file 1 [file biomedicines-13-00702-s001.zip › Supplementary 1 figures edited version.pdf]

Supplementary Table 1- MASTER scale

Author, year	Q1	Q2	Q3	Q4	Q5	Q6	Q7	Q8	Q9	Q10	Q11	Q12	Q13	Q14	Q15	Q16	Q17	Q18	Q19	Q20	Q21	Q22	Q23	Q24	Q25	Q26	Q27	Q28	Q29	Q30	Q31	Q32	Q33	Q34	Q35	Q36	MASTER scale
**Sandborn,2008**	1	1	1	1	1	1	1	0	1	1	1	1	1	1	1	0	0	1	1	1	1	1	1	1	1	1	1	1	1	1	1	1	1	1	1	1	33
**Schreiber,2023**	1	1	1	1	1	1	1	0	1	1	1	1	1	1	1	1	0	1	0	1	1	1	1	1	1	1	1	1	1	1	1	1	0	1	1	1	32
**Sandborn,2022**	1	1	1	1	1	1	0	1	1	1	1	1	1	1	1	0	0	0	1	1	1	1	1	1	1	1	1	1	1	1	1	1	1	1	1	1	32
**NCT02574637**	1	1	1	1	0	1	1	1	1	1	1	1	1	1	1	0	0	1	1	1	1	1	1	1	1	1	1	1	1	1	1	1	1	1	1	1	33
**Sands,2021**	1	1	1	1	1	1	1	1	1	1	1	1	1	1	1	1	0	1	1	1	1	1	1	1	1	1	1	1	1	1	1	1	1	1	1	1	35
**NCT03104413**	1	1	1	1	1	1	1	1	1	1	1	1	1	1	1	1	0	1	1	1	1	1	1	1	1	1	1	1	1	1	1	1	1	1	1	1	35
**NCT03105128**	1	1	1	1	1	1	1	1	1	1	1	1	1	1	1	1	0	1	1	1	1	1	1	1	1	1	1	1	1	1	1	1	1	1	1	1	35

Supplementary Table 2- GRADE Rating for main outcomes

Outcome/ GRADE component	Induction of remission	Endoscopic response	Primary Safety outcome (Adverse events)	Secondary Safety outcomes		
				**Serious adverse events**	**Treatment discontinuation**	**Mortality**
**Overall effect size**	Lower odds of inducing remission with subcutaneous compared to intravenous route (OR 0.77, 95%CI 0.53-1.12).	Lower odds of achieving endoscopic response with subcutaneous compared to intravenous route (OR 0.73, 95%CI 0.46-1.15)	Lower odds of adverse events with subcutaneous compared to intravenous route (OR 0.91, 95%CI 0.63-1.32)	Lower odds of serious adverse events with subcutaneous compared to intravenous route (OR 0.97, 95%CI 0.61-1.53)	Almost similar odds of discontinuation with subcutaneous compared to intravenous route (OR 1.06, 95%CI 0.67-1.68)	Higher odds of mortality with subcutaneous compared to intravenous route (OR 1.17, 95%CI 0.27-5.20)
**Consistency**	GRADE was not downgraded due to no inconsistency and no-to-low heterogeneity $I^2 =$ 0.0%.	GRADE was not downgraded due to no inconsistency and no-to-low heterogeneity I^2 $=$ 0.0%.	GRADE was not downgraded due to no inconsistency and low heterogeneity $I^2 =$ 39%	GRADE was not downgraded due to no inconsistency and no-to-low heterogeneity $I^2 =$ 0.0%.	GRADE was not downgraded due to no inconsistency and no-to-low heterogeneity $I^2 =$ 0.0%.	GRADE was not downgraded due to no inconsistency and no-to-low heterogeneity I^2 $=$ 0.0%.
**Directness**	Participants, interventions, and outcomes were directly relevant to those of interest.	Participants, interventions, and outcomes were directly relevant to those of interest.	Participants, interventions, and outcomes were directly relevant to those of interest.	Participants, interventions, and outcomes were directly relevant to those of interest.	Participants, interventions, and outcomes were directly relevant to those of interest.	Participants, interventions, and outcomes were directly relevant to those of interest.
**Study quality**	Most studies received high-quality scores on the MASTER scale.	Most studies received high-quality scores on the MASTER scale	Most studies received high-quality scores on the MASTER scale.	Most studies received high-quality scores on the MASTER scale.	Most studies received high-quality scores on the MASTER scale.	Most studies received high-quality scores on the MASTER scale.

**Publication bias**	Major asymmetry suggested by the Doi plot confirmed by the LFK index = 4.20.	No asymmetry suggested by the Doi plot confirmed by the LFK index = -0.65.	No asymmetry suggested by the Doi plot confirmed by the LFK index = 0.47.	Minor asymmetry suggested by the Doi plot confirmed by the LFK index = 1.28	Major asymmetry suggested by the Doi plot confirmed by the LFK index = -2.44	No asymmetry suggested by the Doi plot confirmed by the LFK index = 1.
**Final Grade rating**	Moderate level certainty of evidence.	High level certainty of evidence.	High level certainty of evidence.	High level certainty of evidence.	Moderate level certainty evidence due to major asymmetry.	High level certainty of evidence.

Supplementary Table 4- Reasons for treatment discontinuation

Author, year	Subcutaneous discontinuation	Subcutaneous reasons + specified no. if mentioned	Intravenous discontinuation	Intravenous reasons + specified no. if mentioned
Sandborn,2008	13	- 6 Withdrawal by Subject<br>- 3 lost to follow up<br>- 4 other reasons	9	- 4 Withdrawal by Subject<br>- 3 lost to follow-up<br>- 2 other reasons
Schreiber,2023	30	- 11 Withdrawals by Subject<br>- 2 lost to follow-up<br>- 17 other reasons	26	- 7 Withdrawal by Subject<br>- 2 lost to follow-up<br>- 1 death<br>- 16 other reasons
Sands,2021	5	- 1 due to AE<br>- 4 other reasons	8	- 1 due to AE<br>- 7 other reasons
Sandborn,2022	0	-	6	- 2 due to AE<br>- 4 other reasons
NCT02574637	1	Study Terminated By Sponsor	0	
NCT03104413	0		0	
NCT03105128	0		0	

Supplementary Figure 1- Quality assessment of included studies using the MASTER scale.

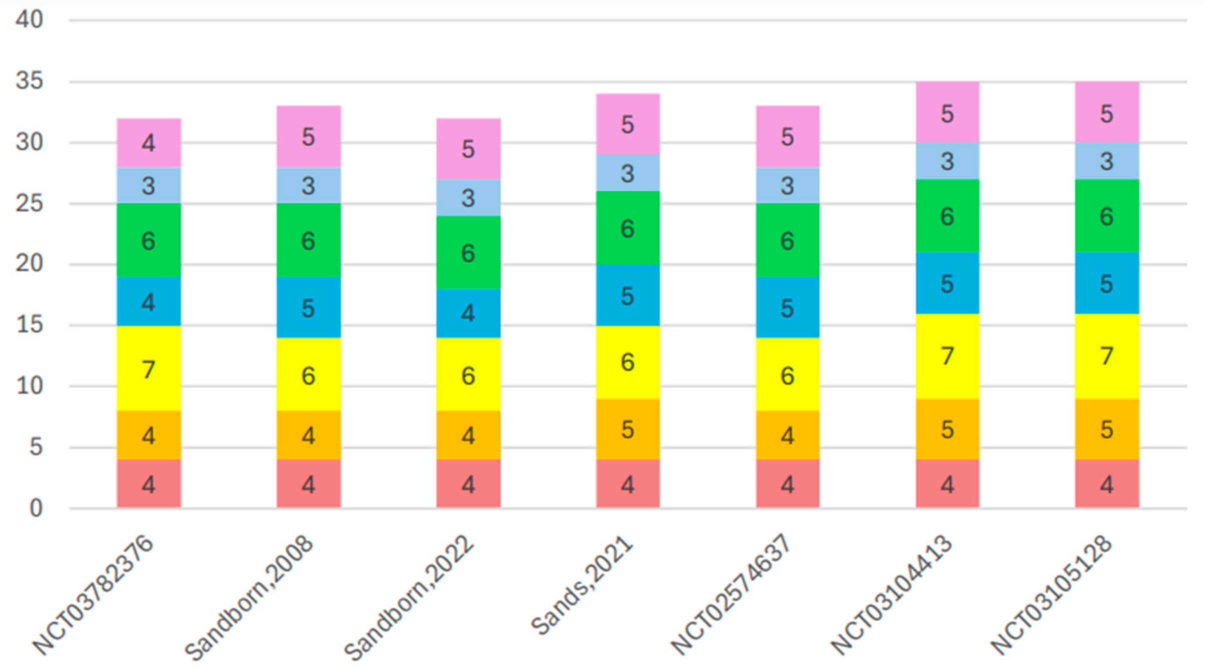

Supplementary Figure 1: *Equal Recruitment **(brown)** has 4 safeguards, Equal Retention (**dark green**) has 5 safeguards, Equal Ascertainment (**sky blue**) has 7 safeguards, Equal Implantation (**purple**) has 6 safeguards, Equal Prognosis (**light green**) has 6 safeguards, Sufficient Analysis (**blue**) has 3 safeguards, Temporal Precedence has 5 safeguards (**yellow**).*

Supplementary Figure 2- DOI plot for induction of remission

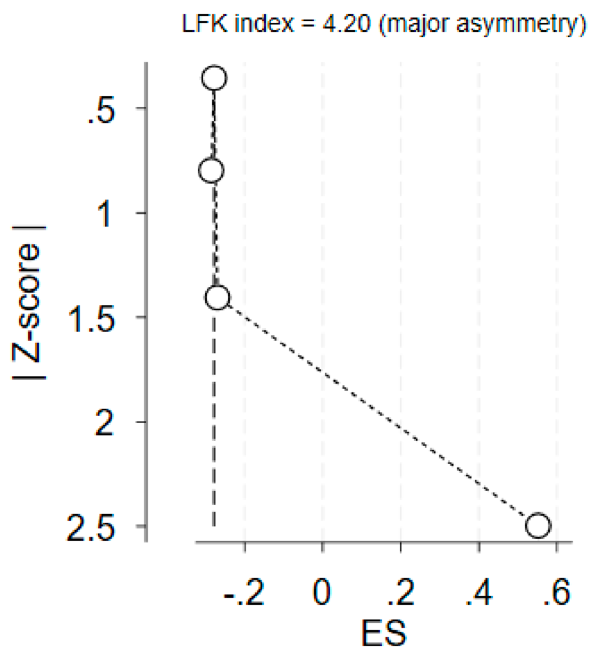

Supplementary Figure 2: *The Doi plot shows major asymmetry, confirmed by the LFK index = 4.20, indicating asymmetry and that publication bias is more likely to be present.*

Supplementary Figure 3- Galbraith plot for induction of remission

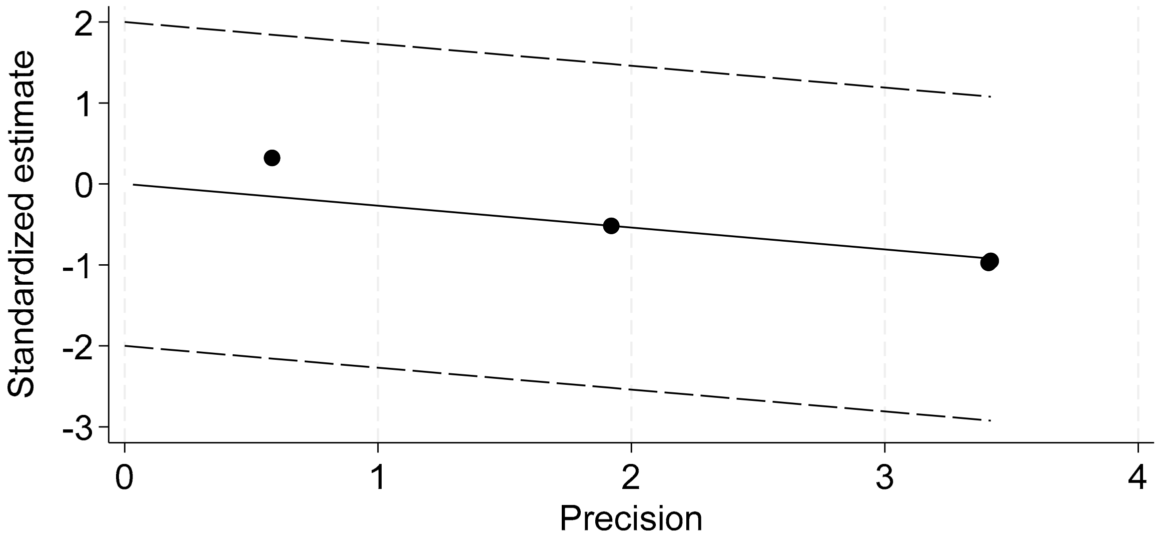

Supplementary Figure 3: *The Galbraith plot shows the four studies that reported the induction of remission at the highest timepoint of the study, one study is overlapping with another study. The regression line showed an effect near the null. All studies, fall within the 95% CIs (the dashed lines) suggesting no heterogeneity.*

Supplementary Figure 4 - Sensitivity analysis for the induction

A. Leave-one-out analysis

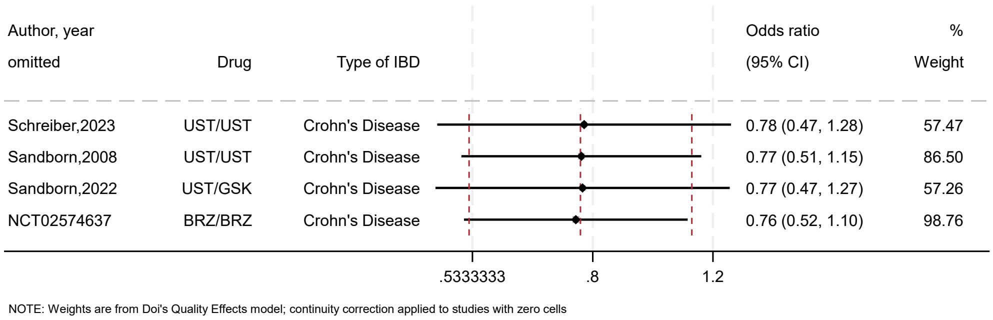

B. *Analysis restricted to Ustekinumab Only*

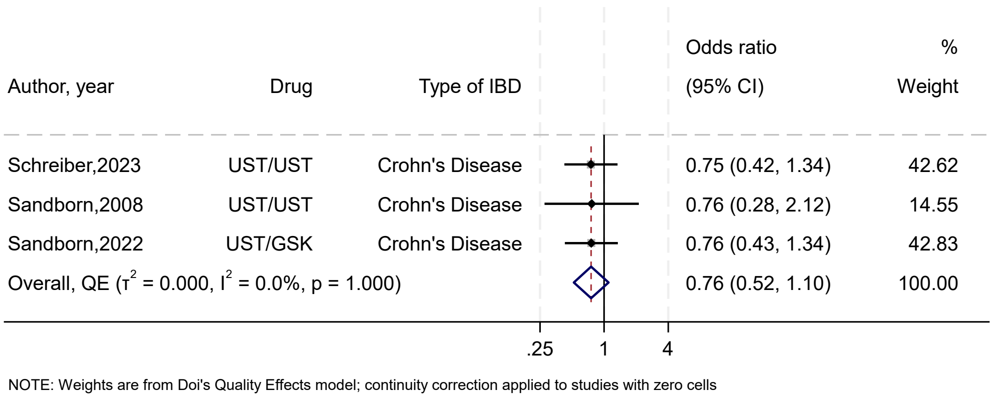

Supplementary Figure 4: *The forest plot shows the Sensitivity analysis for (A) the induction of remission to examine the influence of each study on the meta-analytic outcome estimate(A). (B) shows the induction of remission with Ustekinumab only. The results support those of the main outcome analysis of inducing and maintaining remission. Abbreviations: Ustekinumab (UST), Guselkumab (GSK), Brazikumab (BRZ).*

Supplementary Figure 5- Descriptive forest plot for maintenance of CD remission

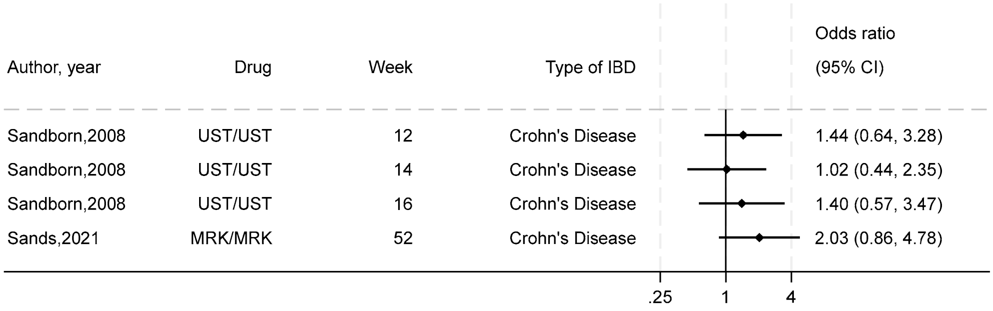

Supplementary Figure 5: *This forest plot shows the results of two trials that compared maintenance of CD remission between subcutaneous and intravenous routes. The first trial measured outcomes at three time-points (12, 14 and 16 weeks). The study results were not meta-analysed because data from the different weeks for Sandborn 2008 came from the same participants. Abbreviations: Ustekinumab (UST), Mirikizumab (MRK).*

Supplementary Figure 6- Forest plot for endoscopic response

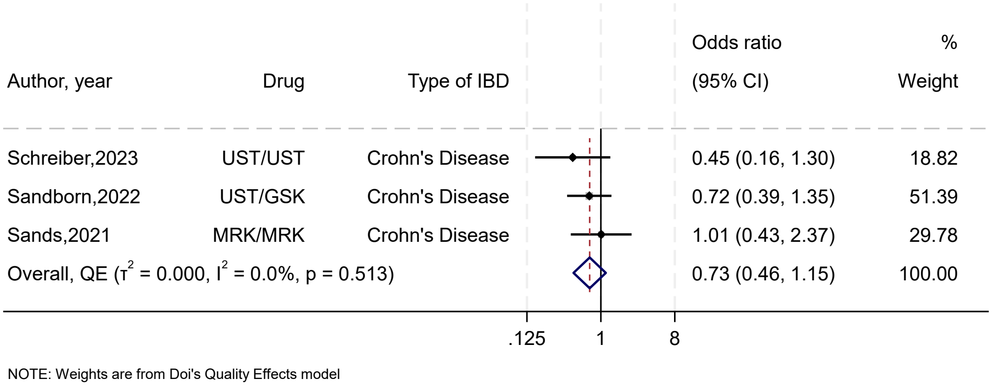

Supplementary Figure 6*: The forest plot shows the results of trials that compared the subcutaneous to the intravenous routes in achieving endoscopic response.*

Abbreviations: Ustekinumab (UST), Guselkumab (GSK), *Mirikizumab (MRK).*

Supplementary Figure 7- Doi plot for endoscopic response

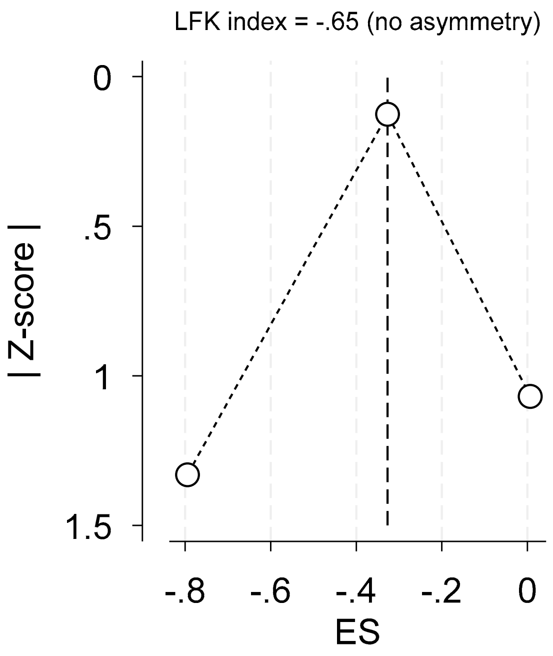

Supplementary Figure 7*: The Doi plot shows no asymmetry, confirmed by the LFK index = -0.65 indicating no publication bias.*

Supplementary Figure 8- Descriptive forest plot for endoscopic remission

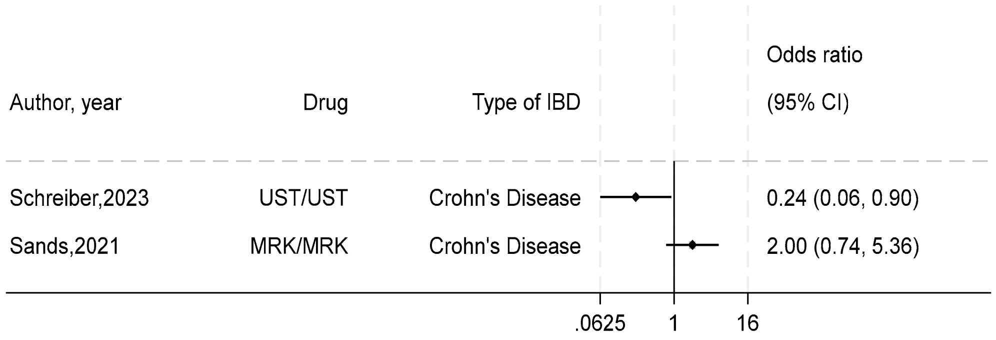

Supplementary Figure 8: *This forest plot shows the results of two trials that compared endoscopic remission of CD between subcutaneous and intravenous routes. Mirikizumab shows two-fold increase in the odds, while Ustekinumab shows 76% decrease in the odds of endoscopic remission. Abbreviations: Ustekinumab (UST), Mirikizumab (MRK).*

Supplementary Figure 9- Doi plot for developing adverse events

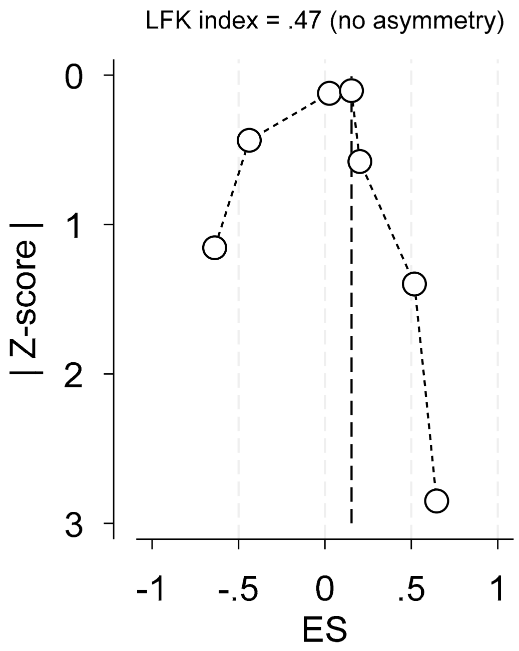

***Supplementary Figure 9**: The Doi plot shows no asymmetry, confirmed by the LFK index = 0.47 indicating no publication bias.*

Supplementary Figure 10- Doi plot for developing serious adverse events

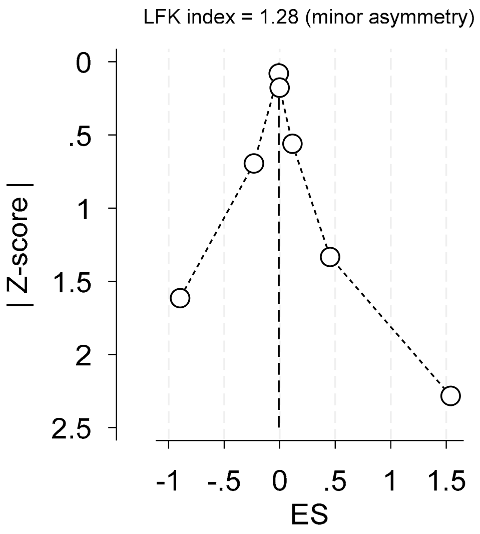

***Supplementary Figure 10**: The Doi plot shows minor asymmetry, confirmed by the LFK index =1.28 suggesting publication bias is less likely.*

Supplementary Figure 11- Doi plot for drug discontinuation

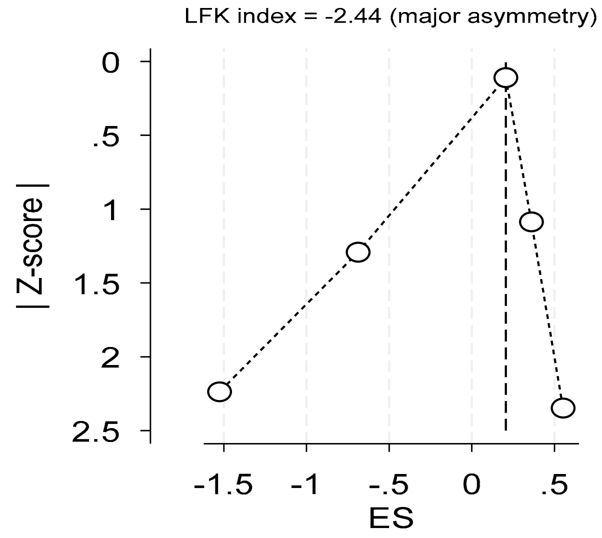

Supplementary Figure 11*: The Doi plot shows major asymmetry, confirmed by the LFK index =-2.44 suggesting presence of publication bias.*

Supplementary Figure 12- Doi plot for mortality

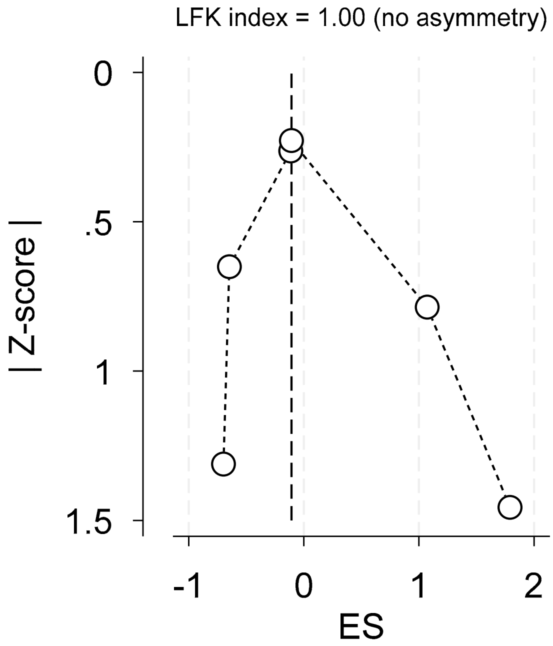

Supplementary Figure 12: *The Doi plot shows no asymmetry, confirmed by the LFK index =1.00 suggesting absence of publication bias.*
